# Modulation of Immunoglobulin Production by Invariant Vα19-Jα33 TCR-Bearing Cells

**DOI:** 10.1371/journal.pone.0020915

**Published:** 2011-06-16

**Authors:** Michio Shimamura, Yi-Ying Huang, Hiroshi Hidaka

**Affiliations:** 1 Tsukuba Research Center for Interdisciplinary Materials Science and Graduate School of Pure and Applied Sciences, University of Tsukuba, Tsukuba, Ibaraki, Japan; 2 Developmental Immunology Unit, Mitsubishi Kagaku Institute of Life Sciences, Machida, Tokyo, Japan; 3 Foundation for Advancement of International Science, Tsukuba, Ibaraki, Japan; Oklahoma Medical Research Foundation, United States of America

## Abstract

We have previously shown that invariant Vα19-Jα33 TCR^+^ (Vα19*i* T) cells suppress the disease progress in some models for organ specific autoimmune diseases and type IV allergy that deteriorate along with decline to excess in Th1- or Th17- immunity. In this study, we examined the effects of over-generation of Vα19*i* T cells on the Th2-controlled immunoglobulin isotype production in the models for type I allergy. IgE production by invariant Vα19-Jα33 TCR transgenic (Tg) mice was suppressed compared with that by non-Tg controls following administration with goat anti-mouse IgD antiserum or OVA, while IgG2a production was not influenced by the introduction of the transgene into the recipients. IgE production by wild type mice was similarly reduced when they were subjected to adoptive transfer with invariant Vα19-Jα33 TCR Tg^+^ but not Tg^−^ cells prior to immunization. Furthermore, the suppression of IgE production by these recipients was enhanced when they were previously administered with a Vα19*i* T cell activator, one of the modified α-mannosyl ceramides. In summary, it is suggested that Vα19*i* T cells have potential to participate in the homeostasis of immunity and that they suppress disease progression resulting from not only Th1- but also Th2- immunity excess.

## Introduction

The TCR α chain consisting of Vα7.2-Jα33 in humans [1] and Vα19-Jα33 (conventionally known as Jα26) in mice [2] is a second type of invariant TCR α chain first found from blood T cells by quantitative PCR analyses. This invariant TCR α chain was preferentially expressed by NK1.1^+^ T but not NK1.1^−^ T cells in the livers of CD1^-/-^ mice where the development of invariant Vα14-Jα18 TCR^+^ cells was suppressed [3]. As the invariant Vα19-Jα33 TCR is frequently detected in the mucosal-associated lymphoid tissues such as gut lamina propria, cells expressing the invariant Vα19-Jα33 TCR are often called as mucosal-associated invariant T (MAIT) cells [4]. Development of invariant Vα19-Jα33 TCR^+^ (Vα19*i* T) cells is dependent on MHC-related protein 1 (MR1) [4] which is an evolutionarily conserved MHC-class Ib molecule [5]. They are selected by bone marrow-derived MR1^+^ hematopoietic cells in the thymus and expand in the periphery interacting with the MR1^+^ B cells [6]. Characterization of mice that over-expressed the invariant Vα19-Jα33 TCR α transgene (Tg) via a natural TCR α promoter revealed that invariant Vα19-Jα33 TCR Tg^+^ cells are distributed to not only gut lamina propria but also the lymphoid organs including the liver of the Tg mice [7]–[9].

Vα19*i* T cells produce immunoregulatory cytokines in response to TCR engagement [7]–[10]. Vα19*i* cells show either Th1- or Th2- biased profiles of immunoregulatory cytokine production depending on the duration and intensity of TCR stimulation in vitro [10], suggesting their involvement in the regulation of the immune system. In fact, NK1.1^+^ Vα19*i* T cells induced IL-10 production from B cells and suppressed the disease progress of experimental autoimmune encephalomyelitis, an animal model of multiple sclerosis [11]. Furthermore, we have recently found that onset of diabetes in NOD mice and induction of delayed-type hypersensitivity toward sheep erythrocytes in mice are suppressed by the over-expression of invariant Vα19-Jα33 TCR α Tg in the subjects [12]. In this study, the effects of the over-generation of Vα19*i* T cells on disease progress in the models for type I allergy were explored to elucidate their immunoregulatory potential.

## Materials and Methods

### Mice

C57BL/6 mice were purchased from Sankyo Service Co. (Tokyo, Japan). CD1-deficient mice were provided by Dr. M.J. Grusby (Harvard University) [13]. They were backcrossed with C57BL/6 mice 6 times, and mice with the phenotypes H-2^b^, NK1.1^+^, and CD1^-/-^ were selected. TCR Cα-deficient mice, that had been backcrossed with C57BL/6 mice for more than 10 generations [14], were donated by Drs. H. Ishikawa (Keio University) and M. Nanno (Yakult Co.). Invariant Vα19-Jα33 TCR transgene cloned from a hybrid line (NB 403, [3]) was linked with TCR α promoter and enhancer and transgenic mouse lines with C57/BL/6, TCR α^-/-^ and CD1^-/-^ genetic backgrounds were established as described previously [9]. All the experiments using mice have been done with the approval of the animal experiment committee of Mitsubishi Kagaku Institute of Life Sciences (the approval No. 105 in 2008).

### Cell preparations

Mononuclear cells (MNC) were prepared from mouse organs by density gradient centrifugation using Lymphosepar II (IBL, Gunma, Japan, *d* = 1.090) for spleen cells or Percoll (Pharmacia, Uppsala, Sweden) for liver cells as described previously [15].

### Administration of mice with allergens and determination of serum immunoglobulin

Mice (8∼12 weeks of age) were intraperitoneally injected with 200 µl of goat anti-mouse IgD antiserum (obtained from Dr. F. Finkelman, University of Cincinnati Medical Center). In other experiments, mice were injected with 100 µg of ovalbumin (OVA) (Sigma) emulsified in complete Freund' s adjuvant followed by 100 µg of OVA in incomplete Freund' s adjuvant after 2 weeks. In some cases, C57BL/6 mice were subjected to adoptive transfer of liver MNCs prepared from Vα19Tg^+^TCR α^-/-^ or C57BL/6 mice (1×10^7^/animal), and after three days these mice were used as recipients. The serum levels of immunoglobulin isotypes and cytokines were determined by ELISA using specific antibodies obtained from BD Bioscience (Pharmingen, San Diego, US). OVA-specific immunoglobulin isotypes were determined as previously reported by Zhang *et al.* [16]. Pooled serum of OVA-immunized C57BL/6 mice was used as a standard and assigned values of OVA-specific IgE, IgG1 and IgG2a of 10 U/ml, 2000 U/ml and 10 U/ml, respectively.

### Cell culture

Mice were immunized with OVA as described above. Spleen MNCs were prepared from them 5 weeks after initial immunization with OVA. They were cultured at the concentration of 5×10^6^ /ml in DMEM containing 10% FCS, 50 µg/ml streptomycin, 50 U/ml penicillin in the presence or absence of OVA (100 µg/ml). Immunoglobulin isotypes and cytokines in the culture supernatants were determined by ELISA on 1 and 3 day of culture.

### Glycolipids

*N*-[1-(α-mannosyl oxymethyl)-3-(4-octyl-phenyl) propyl] hexadecanamide (α-ManCer4Ph) prepared as described previously [17] was provided by Dr. Tadashi Mishina (Mitsubishi Pharma Co.). α-ManCer4Ph was dissolved with DMSO (10 mg/ml). The stock solution was diluted with PBS (x200), sonicated and injected into mice (25 µg/animal) in some experiments.

### Statistical analysis

Data are shown as the mean ±s.d. The significance of differences was determined by the Student's *t*-test.

## Results

### Serum immunoglobulin levels in Vα19 Tg mice

We have shown that Vα19*i* T cells have potential to produce different kind of immunoregulatory cytokines in response to TCR engagement and that over-generation of Vα19*i* cells T suppress the disease progress in the models where disease becomes serious with excess in Th1 or Th17 immunity. In this study we examined the possible involvement of Vα19*i* T cells in the control of serum immunoglobulin isotype levels in animal models for type I allergy.

**Figure 1:**
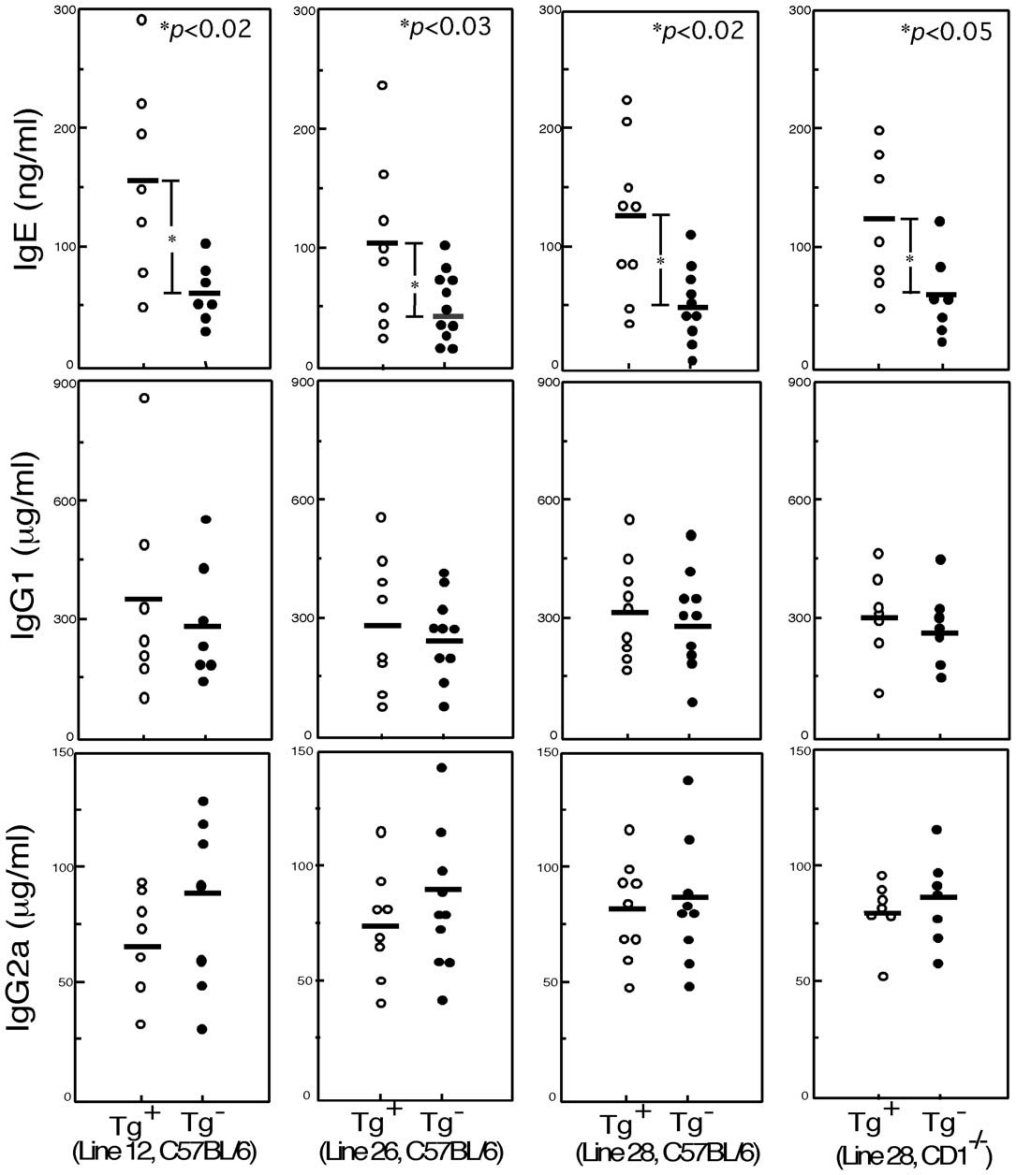
Immunoglobulin isotype levels in the serum of Vα19-Jα33 TCR α (Vα19 ) Tg and non-Tg mice. Serum IgE, IgG1 and IgG2a levels of invariant Vα19 Tg of independently established Tg lines (Line 12, 26, 28) were compared with those of non-Tg mice the same genetic background (C57BL/6 or CD1^-/-^). Each circle represents the immunoglobulin level of an individual mouse (2–3 months-old). The mean levels of each isotype are shown with bars. *P* values in the Student' s *t*-test are indicated in the panels.

The basal levels of several immunoglobulin isotypes in serum were measured in invariant Vα19-Jα33 Tg mice and compared with those in non-Tg mice with the same genetic background (Figure 1). The levels of the Th2-controlled immunoglobulin isotypes (IgE, IgG1) in the Vα19 Tg mice were raised, whereas the levels of Th1-controlled 1gG2a in the Tg mice tended to decrease. A superiority of Th2-controlled isotype production was found in the Vα19-Jα33 Tg mice of independently established lines (line 12, 26 and 28, C57BL/6 background) in common in comparison with their non-Tg litter mates. The serum IgE level of Vα19 Tg^+^ mice was similarly higher than that of non-Tg mice from the same litter with the CD1^-/-^ genetic background where invariant Vα14 NKT cell development is suppressed. Thus, Vα19*i* T cells are suggested to contribute to the maintenance of Th1/Th2 homeostasis in a state that is biased toward Th2 under the physiological conditions.

### Immunoglobulin production by Vα19 Tg mice after injection with a polyclonal immune activator

**Figure 2:**
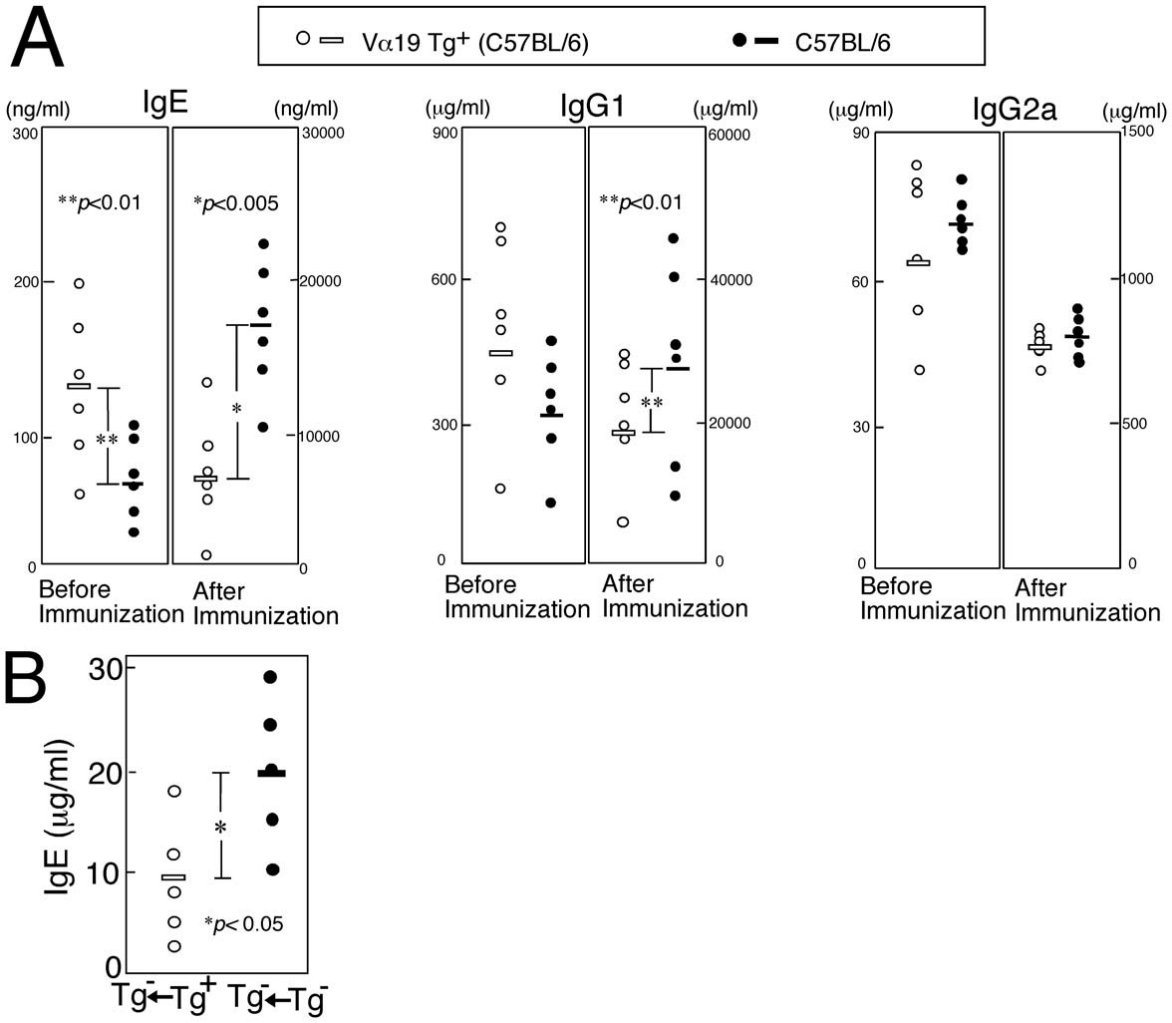
Immunoglobulin isotype levels in the serum of Vα19 Tg and non-Tg mice before and after administration with goat anti-mouse IgD antiserum. (A) Vα19 Tg and non-Tg mice (C57BL/6 genetic background) were injected with a polyclonal immune activator (goat anti-mouse IgD antiserum). Immunoglobulin isotype levels in the serum before and after immunization (1 w) were determined by ELISA. (B) C57BL/6 mice were subjected to adoptive transfer with liver MNCs prepared from either V 19 Tg+ TCR -/- or non-Tg (C57BL/6) mice. After 3 days these mice were immunized with goat anti-mouse IgD antiserum. Serum IgE levels were determined after 1 w. The mean levels are shown with bars.

Immunoglobulin production by Vα19 Tg mice was compared with the production by non-Tg mice after administration with a polyclonal immune activator goat anti-mouse IgD antiserum (Figure 2A). Interestingly, the rise in the serum IgE level in the Vα19 Tg^+^ mice was significantly suppressed (6900±3800 ng/ml) compared with that in non-Tg mice with the same genetic background (C57BL/6) (17300±4300 ng/ml). Similarly, the rise in the serum IgG1 level in the Tg mice (19000±4000 µg/ml) was less that that in the non-Tg mice (26000±6000 µg/ml). Since the rise in IgG2a in the Tg mice (730±70 µg/ml) was comparable to the rise in the non-Tg mice (680±150 µg/ml), the suppressed production of Th2 immunoglobulin isotypes (IgE, IgG1) in the Tg mice may be due to the over-generated Vα19*i* T cells but not to the restriction of the generation of helper T cell repertoires.

To address this issue we compared immunoglobulin production by wild type mice previously subjected to adoptive transfer with lymphocytes prepared from either Vα19 TCR Tg^+^ or non-Tg mice (Figure 2B). Serum IgE levels in the mice subjected to transfer with the Tg^+^ cells were lower than the levels in the mice subjected to transfer with the non-Tg cells. Thus, these results further support the notion that the suppressed production of Th2 immunoglobulin isotypes in the Tg mice is caused by the functions of Vα19*i* T cells rather than by the artificial effects accompanied with the transgene expression.

### Immunoglobulin production by Vα19 Tg mice after immunization with OVA

**Figure 3:**
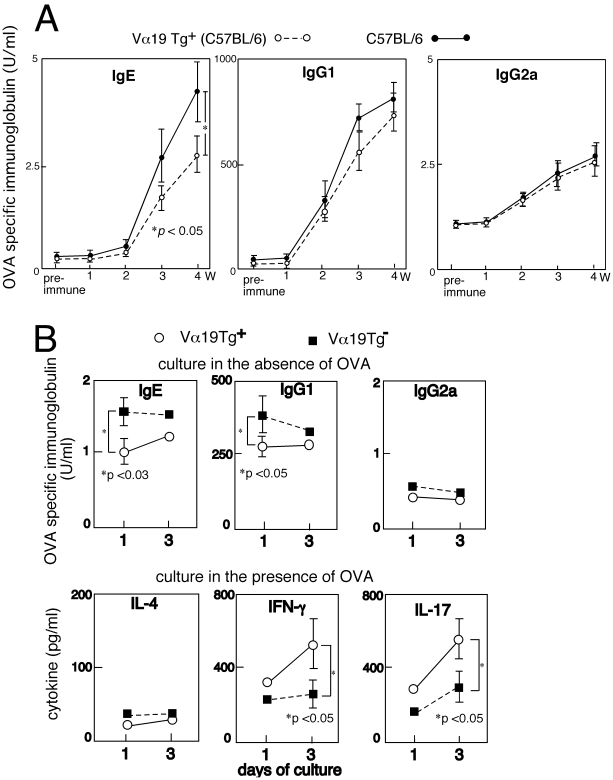
Antigen specific immunoglobulin levels in the serum of Vα19 Tg^+^ and non-Tg mice after immunization with OVA. (A) Vα19Tg and non-Tg mice (C57BL/6 genetic background) were immunized with OVA (at week 0 and 2) as described in Materials and Methods, and OVA-specific immunoglobulin levels in the serum were determined every week by ELISA. The mean ± s.d. of five mice from each strain is shown. Experiments were repeated twice, and essentially similar results were obtained. (B) Immunoglobulin and cytokine production by spleen cells in vitro prepared from OVA-immunized mice. Vα19 Tg^+^ and non-Tg mice were immunized with OVA as shown in (A), and spleen MNCs were prepared from each mouse 5 weeks after initial immunization. The cells were cultured in the presence or absence of OVA, and immunoglobulin isotypes and cytokines in the culture supernatants were determined by ELISA on 1 and 3 day of culture. Mean values of four mice in each group are shown.

**Figure 4:**
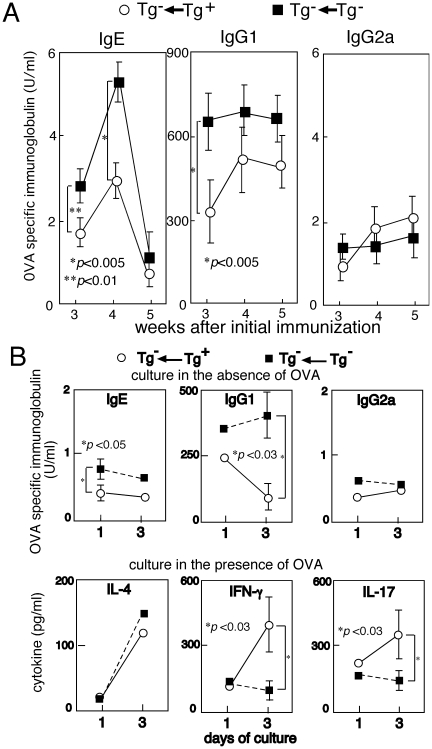
Suppression of OVA specific immunoglobulin production in the mice subjected to adoptive transfer with Vα19 Tg^+^ cells. (A) C57BL/6 mice transferred with liver MNCs isolated from either Vα19 Tg^+^ TCR α^-/-^ or C57BL/6 mice were immunized with OVA as described in Figure 3 and the serum levels of OVA-specific immunoglobulin were determined by ELISA. The mean values obtained from five mice are shown. (B) Immunoglobulin and cytokine production by spleen cells in vitro prepared from OVA-immunized mice. C57BL/6 mice were injected with either Vα19 Tg or non-Tg liver MNCs, and then immunized with OVA as described in (A). Spleen MNCs were prepared from each mouse 5 weeks after initial immunization and the cells were cultured in the presence or absence of OVA. After 1 and 3 day of culture, immunoglobulin isotypes and cytokines in the culture supernatants were determined by ELISA. Mean values of five mice in each group are shown.

Next, antigen-specific immunoglobulin production by Vα19 Tg^+^ and non-Tg mice was compared. Mice were immunized with OVA and the serum levels of OVA-specific immunoglobulin isotypes were determined. OVA-specific IgE level in the serum of the Vα19 Tg mice was lower than that in non-Tg mice with the same background (C57BL/6); whereas, the OVA-specific IgG2a level was comparable between the Tg and non-Tg mice (Figure 3A). Similarly, serum IgE and IgG1 levels in the wild type mice previously subjected to transfer with the Tg^+^ cells were lower than the levels in the mice subjected to transfer with the non-Tg cells, while serum IgG2a levels in the mice injected with Tg^+^ and non-Tg cells were comparable (Figure 4A).

Less production of Th2 controlled immunoglobulin isotypes in the OVA-immunized mice with over-generation of Vα19*i* T cells was also suggested from analysis of the cells isolated from those mice in vitro. The splenocytes prepared from OVA-immunized mice were moved to culture and immunoglobulin isotypes and cytokines in the culture supernatants were determined (Figure 3B, 4B). Production of IgE and IgG1 by the splenocytes from the Tg^+^ mice (Figure 3B) or the Tg^−^ mice transferred with Tg^+^ cells (Figure 4B) was less than that by the splenocytes from the non-Tg mice or non-Tg mice transferred with non-Tg cells, while IgG2a production was comparable between the cells of Tg^+^ and Tg^−^ mice. Interestingly, the splenocytes isolated from the Tg^+^ mice or the Tg^−^ mice transferred with Tg^+^ cells produced more IFN-γ and IL-17 than the splenocytes from the non-Tg mice or the non-Tg mice transferred with non-Tg cells. In contrast IL-4 production by the splenocytes of each origin was comparable. Presumably, Vα19*i* T cells over-generated in the Tg mice participated in the increased production of IFN-γ and IL-17 and eventually brought about the less production of the Th2-controlled immunoglobulin isotypes.

Collectively, these findings suggest that Vα19*i* T cells contribute to the homeostasis of the Th1/Th2 balance in the mice immunized with antigens capable of inducing type I allergy.

### Effects of administration of Vα19*i* T cell activators on the immunoglobulin isotype production

**Figure 5:**
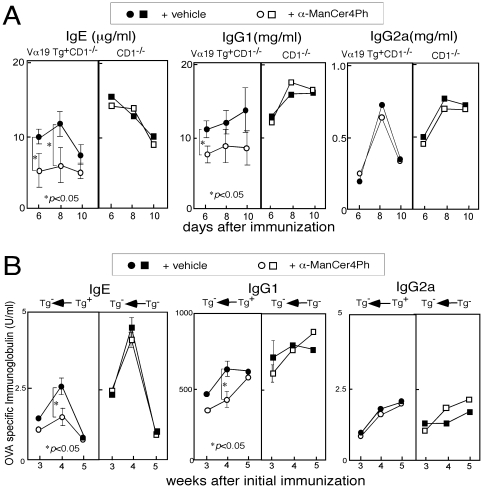
Effects of glycolipid administration on the immunoglobulin production. (A) Vα19 Tg^+^ or Tg^−^ mice with CD1^-/-^ genetic background were injected with goat anti-mouse IgD antiserum. A Vα19*i* T cell activator α-ManCer4Ph was intravenously injected concomitantly with the antigen into a group of mice. Serum levels of immunoglobulin isotypes were determined on day 6, 8 and 10 day by ELISA. Mean values of three mice in each group are shown. (B) C57BL/6 mice subjected to adoptive transfer with liver MNCs from either Vα19 Tg^+^ TCR α^-/-^ or C57BL/6 mice were immunized with OVA as shown in Figure 4. A Vα19*i* cell activator α-ManCer4Ph was intravenously injected concomitantly with OVA into a group of mice (at 0 and 2 w). Serum levels of OVA-specific immunoglobulin isotype were determined after 3, 4 and 5 week by ELISA. Mean values of five mice in each group are shown.

We have previously reported that Vα19*i* T cells are specifically activated with certain α-mannosyl glycolipids in the context of MR1 [18], [19]. A derivative of α-ManCer (α-ManCer4Ph) has potential to induce immunoregulatory cytokine production from Vα19*i* T cells not only in culture but also *in vivo* [18]. We examined the effects of this glycolipid on the immunoglobulin isotype production.Invariant TCR α Tg^+^ or Tg^−^ mice with CD1^-/-^ genetic background were injected with α-ManCer4Ph concomitantly with goat anti-mouse IgD antiserum, and immunoglobulin isotypes in the serum were determined (Figure 5A). IgE and IgG1 but not IgG2a production was reduced in the Tg^+^ mice and the reduction was enhanced with the α-ManCer4Ph administration. Next, invariant TCR α Tg^−^ mice subjected to adoptive transfer with either invariant Vα19 TCR Tg^+^ or Tg^−^ cells were injected with α-ManCer4Ph concomitantly with immunization with OVA (at 0 and 2 week), and OVA-specific immunoglobulin isotypes in the serum were determined (Figure 5B). The reduction in the Th2 controlled immunoglobulin isotype production was similarly observed in the Tg^−^ mice transferred with Tg^+^ but not with Tg^−^ cells when the mice were injected with α-ManCer4Ph. Thus, Vα19*i* T cells activated with α-ManCer4Ph are likely to work as a regulator of immunoglobulin isotype production.

## Discussion

We have previously found that Vα19*i* T cells promptly produce immunoregulatory cytokines upon invariant TCR engagement and that the cytokine spectra are altered according to the intensity and the duration of stimulation to invariant TCR [10]. We speculate that the regulatory functions of Vα19*i* T cells arise from their potential to produce either Th1 or Th2 -dominant cytokines according to the circumstances.

The serum levels of Th2-controlled immunoglobulin isotypes (IgE and IgG1) in the invariant Vα19-Jα33 TCR Tg mice are higher than those in the non-Tg mice (Figure 1). Vα19*i* T cells are thus suggested to induce somewhat Th2-biased immunity under the physiological conditions (without administration of antigens). In accordance with this observation, we have previously found the suppressed progression of diseases in the models for organ specific inflammatory autoimmunity [11], [12] and T cell mediated type IV allergy [12] where excess in Th1 immunity tends to worsen the disease.

On the contrary, the production of IgE, and to some extent IgG1, following administration of allergens was suppressed in the invariant Vα19-Jα33 TCR Tg mice when compared with the production in the non-Tg mice (Figure 2, 3). It is thus supposed that Vα19*i* T cells over-generated in the Tg mice lessened the excess in Th2 immunity with exposure to allergens. The increased production of IL-17 and IFN-γ in culture by the spleen cells of the OVA-immunized Tg^+^ mice or non-Tg mice transferred with invariant Vα19-Jα33 TCR Tg^+^ cells may account for the restoration from the Th2 excess in those mice (Figure 3B, 4B). Interestingly, negative regulation of established allergic asthma by IL-17 has been suggested in a recent report [20]. Recently Vα19*i* MAIT cells are demonstrated to participate in the prevention of microbial infection [21], [22]. These cells possibly contribute to the induction of Th1 or Th17 immunity against invading microbes. The potential of Vα19*i* T cells underlying the exclusion of microbes and the restoration from the excess in Th2 immunity during exposure to allergens may be partially in common.

In the present study Vα19*i* T cells are suggested to have the capacity to contribute to the homeostasis of Th1 and Th2 immunity. However, it is not clearly understood yet how Vα19*i* T cells regulate the immune system. Presumably, the affinity of the antigen/MR1 complex to invariant Vα19 TCR definitively influences the pattern of immunoregulatory cytokine production by Vα19*i* T cells judging from our previous observations that the profiles of cytokines are dependent on how invariant Vα19 TCR is stimulated in vitro [10]. It may be possible to speculate that Vα19*i* T cells are induced to produce a slight amount of Th2-dominant cytokines continuously by relatively weak stimulation to the invariant TCR with certain self or foreign antigens when the hosts are under the physiological conditions. On the other hand, invasion of certain foreign pathogens or allergens may induce the hosts to generate putative MR1-coupled antigens with high affinity to invariant Vα19 TCR that are capable of inducing Th1 or Th17-dominant cytokine production from Vα19*i* T cells. The cytokines secreted by Vα19*i* T cells may contribute to the induction of Th1 or Th17 immunity and have crucial roles in the suppression of Th2-controlled immunoglobulin production.

It is interesting that the serum levels of Th2-controlled immunoglobulin isotypes in the invariant Vα14-Jα18 TCR Tg mice are similarly higher than those in the non-Tg mice [23], [24]. Taking into account the report that human invariant Vα24-Jα18 TCR^+^ cells produce altered immunoregulatory cytokines depending on the way of TCR engagement [25], Vα14 NKT cells are also continuously stimulated with certain self or foreign antigens thereby induced to produce Th2-biased cytokines under the physiological conditions. The elevated serum levels of Th2-controlled immunoglobulin isotypes found in such invariant TCR α Tg mice are probably due to the immunoregulatory functions of Vα19*i* T or Vα14 NKT cells, since the serum IgE levels in irrelevant TCR α (Vα8-Jα37) Tg mice are comparable to those in the non-Tg mice [25].

While participation of Vα14 NKT cells in the regulation of Th2-controlled immunoglobulin production in non-primed mice is suggested, the regulatory function by Vα14 NKT cell in mice exposed to allergens is controversial. For instance, Cui *et al*. reported that serum IgE levels in Jα18^-/-^ mice were comparable to those in wild type mice following OVA immunization [26], whereas Akbari *et al*. demonstrated the reduction in the OVA-specific IgE production by Jα18^-/-^ or CD1^-/-^ mice compared with the wild type controls [27]. However, it is likely that the suppression of Th2-controlled immunoglobulin production observed in the invariant Vα19 Tg mice upon exposure to allergens is substantially attributable to Vα19*i* T cells because the suppression was observed even in the Vα19 Tg mice with CD1-deficient genetic background and was enhanced with α-ManCer4Ph administration (Figure 5A).

So far, a synthetic glycolipid α-ManCer4Ph has been demonstrated to induce production of IFN-γ and IL-17 from Vα19*i* T cells in an MR1-dependent manner in vitro and in vivo more intensively than the others, although this glycolipid also has potential to induce production of IL-4 to a degree [18]. We speculate that α-ManCer4Ph might partially mimic the roles of putative natural ligands for MR1-restricted Vα19*i* T cells. Administration of α-ManCer4Ph has been shown to suppress Th2-controlled immunoglobulin production in the Tg^+^ mice and non-Tg mice transferred with Vα19 TCR Tg^+^ cells following antigen immunization (Figure 5). Thus Vα19*i* T cells under the MR1-restriction are likely to take important roles in the regulation of immunoglobulin production. However, MR1-restriction of the regulatory functions by Vα19*i* T cells should be formally verified by the examination of immunoglobulin production by Vα19 Tg^+^ mice under the MR1^+^ and MR1^−^ conditions.
